# Comparison of the sensitivity of *Danio rerio* and *Poecilia reticulata* to silver nitrate in short-term tests

**DOI:** 10.2478/v10102-010-0040-0

**Published:** 2010-11

**Authors:** Petra Doleželová, Stanislava Mácová, Vladimíra Pištěková, Zdeňka Svobodová, Iveta Bedáňová, Eva Voslářová

**Affiliations:** Department of Public Veterinary Medicine and Toxicology, Faculty of Veterinary Hygiene and Ecology, University of Veterinary and Pharmaceutical Sciences, Brno, CZECH REPUBLIC

**Keywords:** Zebra fish, guppy, fish, toxicity, LC_50_, silver, susceptibility

## Abstract

The aim of this study is to assess the acute toxicity of silver nitrate in adult zebra fish and adult guppies and to compare the sensitivity of these species to this compound. Silver is a naturally occurring element in our environment and it combines with other elements such as sulfide, chloride, and nitrate. Silver, in the form of silver nitrate, is one of the most toxic metals affecting freshwater fish. Industry, particularly photographical and electrotechnical, is the major contributor of silver that is released into the environment. Tests of acute toxicity were performed on the most common species of aquarium fish, *Danio rerio* and *Poecilia reticulata*. Both zebra fish and guppies were exposed to progressive concentrations of silver nitrate; a semi-static method according to OECD 203 was used. In each test series, 6 tests of acute toxicity were conducted, with 10 fish used for each separate concentration and for the control group. The results (number of fish deaths in the individual test concentrations) were subjected to probit analysis (EKO-TOX 5.1 software) to determine the 96hLC_50_ AgNO_3_ values. The 96hLC_50_ AgNO_3_ value for the zebra fish was (mean±SEM) 15±0.52 µg/l and for the guppies was (mean±SEM) 17.14±5.43 µg/l. We didn't find any statistically significant difference between the sensitivity of zebra fish and guppies. The results reported in this study are in agreement with LC_50_ values published in peer-reviewed literature, and conclude that AgNO_3_ is one of the most toxic compounds known to fishery.

## Introduction

Ecotoxicological evaluations are required by chemical control regulations in order to classify new substances in relation to their potential hazard to the environment (Gallo *et al*., 1995). Both guppy and zebra fish belong to the eight recommended model organisms in ecotoxicology testing established in the guidelines set by the Organization for Economic Cooperation and Development (OECD). The main advantages of using the zebra fish and the guppies for these tests include their easy availability, economical husbandry, their small yet accessible size, and their high reproductive capacity and genetic tractability. The equivalent susceptibility to chemical compounds is highly desired attribute (Vittozzi and DeAngelis 1991). There are several studies that have observed differences in the sensitivity of some fish species to different chemical compounds (Gallo *et al*., 1995; Vittozzi and DeAngelis 1991; Svobodova and Vykusova 1991). Because of species-specific toxicity, toxicity testing with only one fish species may be inadequate to evaluate chemicals belonging to these classes for their environmental impact (Gallo *et al*., 1995).

Zebra fish (*Danio rerio*) are small (3–4 cm long) tropical freshwater fish that originate from India. They reach sexual maturity at about three months. Females lay hundreds of eggs approximately every other week, a feature that greatly facilitates genetic analysis. Eggs are fertilized externally and embryos are completely transparent, allowing one to follow the development of every individual cell (Driever *et al*., 1994).

Guppies (*Poecilia reticulata*) are small tropical fish, native to the coastal streams of northeast South America. Female guppies are live-bearing. Males fertilize the eggs using a stick-like modified anal fin, the so-called gonopodium. Guppies are ovoviviparous, i.e. the eggs develop inside the mother.

The aim of this study is to assess the acute toxicity of silver nitrate in adult zebra fish and adult guppies and compare their sensitivity to this compound.

Silver is a naturally occurring element that is extensively utilized in the photographic and imaging industry, as well as in electronics and electrical applications. It is known to have been discharged into the environment from its industrial applications, thus, leading to the possibility of exposure to aquatic organisms (Purcell and Peters 1998).

Any exposure scenario regarding silver must take into account its chemical form, because its toxicity is highly dependent on how the chemical is presented to the organism and the availability of the material from a biological standpoint. This phenomenon of metals behaving differently in different chemical forms is known as speciation (Purcell and Peters 1998). Silver is found in the environment combined with other elements such as sulfide, chloride, and nitrate. Silver nitrate is highly toxic to freshwater fish. The toxicity of silver nitrate has been attributed to the presence of Ag^+^, a free ionic silver ion. Other dissolved forms of silver are much less toxic (LeBlanc *et al*., 1984). Accordingly, many processes and water characteristics reduce silver toxicity by stopping the formation of free Ag^+^, binding Ag^+^, or preventing the binding of Ag^+^ to the reactive surfaces of organisms. The solubility of a silver compound, and the presence of complexing ligands such as Cl^–^, dissolved organic carbons and sulfides, plus competing ions such as Na^+^ and H^+^ are important in reducing the bioavailability of Ag^+^ to toxic sites at the gills (Ratte 1999). Ag^+^ can bind to a negatively charged ligand in the gill, and inhibits the Na^+^, K^+^-ATPase located at the baso-lateral membrane of the gill epithelium (Morgan *et al*., 1997).

Assessing the influence of water temperature on silver intake and the elimination of previously accumulated Ag by rainbow trout (*Oncorhynchus mykkis*) showed that higher water temperatures induce greater accumulation of Ag in the gills, plasma and the liver and bile. Regarding elimination, there was no significant difference between warm and cold fish (Nichols and Playle 2004).

Exposure to waterborne AgNO_3_ resulted in a severe disturbance of branchial Na^+^ and Cl^–^ regulation. Fish exposed to Ag^+^ immediately began to lose ions to the surrounding water as a result of a severe, persistent inhibition of branchial influx rates and a lesser, temporary stimulation of branchial efflux rates (Morgan *et al*., 1997). Ag entered the fish and accumulated in blood plasma and in the liver. Other internal disturbances included the decrease of plasma Na^+^ and Cl^–^, metabolic acidosis, the disruption of fluid volume regulation, hemo-concentration, and splenic contraction (Wood *et al*., 1996, Grosell *et al*., 2002).

## Materials and methods

Acute toxicity tests were performed on aquarium fish *Danio rerio* (aged 2–3 months, body length 25±5 mm) and *Poecilia reticulata* (aged 2–3 months, body length 20±5 mm).

Both zebra fish and guppies were exposed to a series of progressive concentrations of silver nitrate (2–50 µg/l); this procedure complied with OECD No. 203 Acute Toxicity Test according to the Fish-Semistatic Method guidelines. The fish were acclimatized for 96 hours. In each test series, 6 tests of acute toxicity were made, with 10 fish used for each concentration and for the control group. Every 48 hours the solution in each concentration was changed. Every 24 hours water temperature, pH, and the oxygen saturation of water were recorded, as well as the fish mortality rate.

The basic physical and chemical indices of the diluted water used in the acute toxicity test were as follows: ANC_4.5_ (acid neutralization capacity) 1.15 mmol/l; COD_Mn_ (chemical oxygen demand) 1.9mg/l; total ammonia bellow limit of determination; NO_3_^–^ 24.5–31.4mg/l; NO_2_^–^ below limit of determination; Cl^–^ 19.1mg/l; sum of Ca+Mg 14mg/l. Water temperatures in the test was 23±1°C, the oxygen saturation of water was above 60% (ranging from 85 to 96%), and pH ranged from 8.04 to 8.66.

The results (number of fish deaths in individual test concentrations) were subjected to a probit analysis (EKO-TOX 5.1 software) to determine the 96hLC_50_ AgNO_3_ values. The statistical significance of the difference between LC_50_ values for the guppies and the zebra fish was evaluated by using a non-parametric Mann-Whitney test and Unistat 5.1 programme.

## Results

The 96hLC_50_ AgNO_3_ value for *Danio rerio* was (mean±SEM) 15±0.52 µg/l and for *Poecilia reticulata* was 17.14±5.43 µg/l. We didn't find any statistical significant difference between the sensitivity of zebra fish and guppies.

## Discussion

Our (and other author's) reported measurements of 96hLC_50_ values suggest that the AgNO_3_ is one of the most toxic metal salts in freshwater.

Hogstrand and Wood (1998) observed the effects of silver (in the form of silver nitrate) in freshwater fish and seawater fish and found out that silver nitrate toxicity was much lower in seawater fish (96hLC_50_ ranged 330–2,700 µg/l) than in freshwater fish (96hLC_50_ ranged 5–70 µg/l).

Davies *et al*., (1978) recorded mean 96hLC_50_ values in rainbow trout of 6.5 µg/l and 13 µg/l in soft and hard water. Similar values were obtained in rainbow trout by Nebeker *et al*., (1983) and Hogstrand *et al*., (1996). In bluegill (*Lepomis macrochirus*), it was higher 60 µg/l (Buccafusco *et al*., 1981). Silver toxicity was also measured in fathead minnows (*Pimephales promelas*) and the 96hLC_50_ values were 6.7 µg/l (Holcombe *et al*., 1983) or 5.6–9.7 µg/l (Nebeker *et al*., 1983). In channel catfish (*Ictalurus punctatus*) it was determined that the 96hLC_50_ value was 17.3 µg/l (Holcombe *et al*., 1983). Thus, LC_50_ values obtained by this study are mostly comparable with previous results.

In this study the acute toxicity of silver nitrate in guppies and zebra fish has been assessed separately with 96h toxicity tests, and there was no significant difference in the sensitivity of zebra fish and guppies to this compound. However, Gallo *et al*., (1995) compared the acute toxicity of two carbamate pesticides, aldicarb and carbaryl, and found out that guppies are more sensitive (the toxicity of both carbamates was higher) than that of zebra fish.

Other authors that have compared acute toxicity between zebra fish and guppy are Svobodova and Vykusova (1991). Their study identified the acute toxicity of the chemical compounds that are commonly used as standards in current toxicity testing (p-nitrophenol, potassium dichromate and zinc sulfate). In the tests for the acute toxicity of p-nitrophenol the values of 48hLC_50_ and 48hLC_5_ were almost the same in both species. Statistically significant higher values of 48hLC_50_ and 48hLC_5_ for zinc sulfate were recorded in the zebra fish. In contrast, acute toxicity tests for potassium dichromate found that zebra fish proved to be more sensitive.

These results may indicate species-specific toxicity, with the conclusion that ecotoxicological tests performed only on one fish species may lead to an erroneous classification of chemical compounds.
